# Evidence-based campaigning

**DOI:** 10.1186/s13690-018-0302-4

**Published:** 2018-10-01

**Authors:** Brian Martin

**Affiliations:** School of Humanities and Social Inquiry, University of Wollongong, Wollongong, NSW 2522 Australia

**Keywords:** Evidence-based medicine, Health promotion, Campaigning, Vaccination, Australia

## Abstract

**Background:**

When promoting public health measures, such as reducing smoking, there are many different approaches, for example providing information, imposing legal restrictions, taxing products, and changing cultures. By analogy with evidence-based medicine, different approaches to campaigning for health promotion can be compared by obtaining evidence of effectiveness. However, evaluating the effectiveness of campaigning approaches is far more difficult than evaluating drugs or medical procedures, because controls are seldom possible, endpoints are difficult to specify, multiple factors influence outcomes, and the targets of campaigns are people or organizations that may resist.

**Methods:**

Ten ideal campaigning types are proposed: positive and negative approaches to the five categories of information, attitude, arguments, authorities and incentives. To illustrate the ideal types and the complexities of evaluating approaches to campaigning, three contrasting Australian strategies to promote vaccination are examined.

**Results:**

Each of the three vaccination-promotion strategies showed the presence of several ideal campaigning types, but with distinct differences in emphasis. With available evidence, it is difficult to assess the relative effectiveness of the three strategies.

**Conclusion:**

Because of the difficulty in obtaining evidence, claims about the effectiveness of general approaches to health promotion should be treated with scepticism, especially when presented by partisans. There are inherent difficulties in making campaigning evidence-based.

## Background

The concept of evidence-based medicine has become a talisman in contemporary medical practice [1, 2]. The idea is that medical interventions should be based on research and not rely primarily on tradition or professional judgement. The evidence can be of various types; most sought after are double-blind randomised clinical trials. Although many medical procedures continue to be undertaken without strong evidence of this sort, the demand to provide evidence has become an important tool for promoting change. However, evidence-based medicine can be subverted, so care is needed to balance the imperatives of evidence with professional judgement [3].

In contrast to the role of evidence within medical practice, campaigning for public health interventions relies much more on intuition and experience, including whether methods appeal to campaigners. A classic case is the anti-smoking movement. The evidence base against smoking was overwhelming to all except tobacco-company apologists, but the evidence base for comparing diverse ways of intervening against smoking was largely absent. Methods used have included publicising research findings, making authoritative announcements (most prominently by the US Surgeon General), restricting cigarette advertisements, banning smoking in specified places, taxing cigarettes, culture jamming (refacing billboards advertising cigarettes), legal actions against tobacco companies, producing anti-smoking advertisements, requiring plain packaging and changing cultural attitudes to smoking [4]. It might be argued that any and all of these interventions are justified by the enormous harm caused by smoking, but it still can be asked, which interventions have been most effective in terms of cost and effort? This is not easy to answer, because interventions seldom can be assessed separately. For example, raising taxes on cigarettes requires information campaigns and lobbying to convince government officials to increase the taxes. Furthermore, interventions were fiercely countered by the tobacco industry.

Effective campaigning can make a huge difference to social welfare. Consider, for example, the issue of crime. Criminologists have produced findings showing that tougher penalties do not reduce the crime rate [5], yet politicians in some countries have ignored this evidence and campaigned on platforms of being “tough on crime.” In the US, the result has been incarceration of millions of people at an expenditure of hundreds of billions of dollars [6]. Another example is the so-called war on drugs, namely enforcing prohibitions against illicit drugs, costing vast amounts of money and stimulating criminal activity apparently without significant impacts on drug use [7–9]. The war on drugs was launched without strong evidence that it was more effective in reducing drug harms than alternatives [10].

There is a fair bit of evidence about the effectiveness of social interventions, for example to reduce crime or improve student performance; some popular interventions turn out to be counterproductive [11]. Evidence-based campaigning sounds worthwhile, but developing a sound evidence base is far more complex than when testing drugs, medical devices or even social interventions. There is a considerable body of writing about campaigning for social change (e.g., [12–15]), but most recommendations are based on experience, not systematic testing [16].

At least five types of complexities need to be addressed.

1. Control groups. Because different sorts of campaigning are undertaken simultaneously, it is usually difficult to find a suitable control group. Occasionally there are de facto controls when a different city or country uses a different approach to campaigning, but cultural or political differences can make comparisons difficult.
2. Outcomes. A choice needs to be made whether short-term or long-term outcomes should be considered. Furthermore, the outcomes or endpoints need to be appropriate. This is hard enough for something like cancer, in which a tumour might shrink but the death rate remains unchanged.
3. Independent assessment. Independent, non-partisan assessments of evidence are needed. Blinding can be used in clinical trials but is not possible when comparing campaigning methods and outcomes.
4. Causality. Multiple causal factors influence outcomes, so determining the factors responsible for changes is difficult.
5. Agency. The targets of campaigning are active agents and may contest, resist or subvert efforts to change their behaviour. This is analogous to the development of resistance to pesticides or antibiotics, except that it can happen immediately. Because people — the targets of campaigns — are conscious agents, campaigning is less like an intervention and more like a game of strategy [17].

Both the difficulties and importance of evaluating campaigning methods are shown by the long-standing debate between campaigners seeking to overthrow repressive governments. Some challengers use the method of armed struggle whereas others use methods of nonviolent action such as rallies, strikes, boycotts and sit-ins. Disagreement between advocates of these two approaches has persisted since at least the days of Gandhi’s early campaigns in India in the 1920s, when his leadership of the independence movement was questioned by Marxists such as M. N. Roy. Proponents of armed struggle and of nonviolent struggle used selected examples to argue their respective cases, but it was not until recently that systematic comparisons were undertaken. Chenoweth and Stephan [18] analysed 323 conflicts over a century and found that nonviolent anti-regime struggles were far more likely to succeed than armed ones. Their research will not end the debate, but for those subscribing to evidence-based campaigning, nonviolence should be the current recommendation for anti-regime struggles. In this case, the implications in terms of lives and freedom are enormous.

To help provide insight into the difficulties of evaluating the effectiveness of different approaches to health campaigning, in the following section ten ideal-type approaches are proposed. In the subsequent sections, application of these ideal types is illustrated using a case study from promotion of vaccination in Australia. Three main approaches to recent vaccination promotion are identified, each involving several ideal types, illustrated using submissions to a government inquiry. Then some of the arguments and evidence for each of the three approaches are examined, illustrating the difficulties in making a definitive judgement about their comparative effectiveness. A key conclusion from this analysis is that evaluating approaches to campaigning is far more challenging than evaluating medical interventions, and therefore scepticism about the claims of advocates is warranted.

## Methods

### Approaches to campaigning

Given the difficulty of evaluating campaigning methods, the aim here is more modest: to classify some approaches to campaigning and highlight some of their assumptions and implications. “Approaches” can be distinguished from “methods” by being more general. For example, advertising is a method — for a comprehensive assessment of evidence, see [19] — within the general approach of publicity; other methods include media stories and social-media promotion. It is usually easier to assess the comparative advantages of alternative methods than to judge which approaches are more effective. Instead of “approaches” and “methods,” it is also possible to talk of “strategies” and “tactics.”

Given that there seems to be no standard way of classifying approaches to campaigning, a framework is proposed here involving ten ideal types, shown in Table 1. This involves five logically distinct categories, each with positive and negative applications.

**Table 1:** Ten approaches to campaigning

*Category*	Positive approach	Negative approach
*Information*	Publicity	Censorship
*Attitude*	Valuing	Devaluing
*Arguments*	Arguments for	Arguments against
*Authorities*	Endorsement	Discrediting
*Incentives*	Rewards	Penalties

In sociology, ideal types are hypothetical categories that capture the essence of phenomena, even though they may never be found in pure form [20]. Ideal types offer a way of better understanding the mixtures that occur in practice.

In the category of information, there are two approaches. The first is publicity about the favoured goal, for example providing information about non-smoking venues or smoking-cessation treatments. The negative approach is censorship, the idea being to prevent access to contrary information, for example by outlawing cigarette advertising. The idea behind censorship is to change the people’s behaviour by preventing them from hearing certain messages.

In the category of attitude, the positive approach is to value favoured individuals or behaviours, as in the slogan “Kiss a non-smoker and taste the difference” or in welcoming attitudes towards non-smokers. The negative approach is to devalue unwelcome individuals or behaviours, for example treating smokers as pariahs. Tobacco companies seek to make smoking seem sophisticated, rebellious, sexual or cool, whereas smoking opponents seek to make it seem dirty, foolish and uncool.

The category of arguments includes evidence and logic used to persuade people to adopt a certain view, and can incorporate scientific, ethical, political, economic and social dimensions. Arguments can either be in favour of a viewpoint or against the contrary viewpoint. In this category sit debates over the health hazards of smoking and second-hand smoke.

The category of authorities includes, on the positive side, endorsements for a practice from governments, scientists, expert panels and others with credibility, and, on the negative side, discrediting anyone with credentials or visibility who takes a contrary viewpoint. In the smoking debate, endorsements of the case against smoking came from scientists and government health departments, while scientists who defended the tobacco industry were sometimes criticised as having conflicts of interest.

The category of incentives operates by encouraging desired behaviour or discouraging undesired behaviour. Lower insurance premiums for non-smokers are a positive financial incentive; taxes on cigarettes discourage consumption.

A special category of incentive is the win-win solution, which involves finding a different path that achieves the desired goal. There are different types of win-win solutions, depending on the goal. If the goal is to end exposure to second-hand smoke, then having separate areas for smokers is one option. If the goal is to reduce smoking, a win-win solution might be measures to encourage sports requiring breath capacity, given that few serious swimmers or runners are smokers.

Interactions between these ideal types are to be expected. For examples, a combination of incentives and persuasion can contribute to cultural change. The value of thinking in terms of ideal types is to identify approaches and, if possible, determine whether they are effective, ineffective or even counterproductive.

To illustrate the importance of campaigning methods, and to show the complexities of assessing evidence about them, a case study from the Australian vaccination debate is examined. Both supporters and critics of vaccination have the same goal, improving children’s health, so it would be possible to study both sides in the debate. However, to make things simpler, only methods for promoting vaccination will be considered, as they illustrate most of the ten ideal types. In the following sections, three distinct approaches to vaccination promotion are described, noting the difficulties in assessing evidence about their effectiveness.

### Promoting vaccination in Australia

The Australian government recommends a schedule of vaccinations for children. Coverage is quite high and stable [21, 22].

There are four main reasons why immunity to vaccine-preventable diseases is not as high as it could be. First, some children cannot or should not receive certain vaccines because of medical conditions, for example due to impaired immune systems. Secondly, vaccines do not trigger immunity in all those who receive them, despite repeat doses. Related to this, vaccine-induced immunity can decline with time for some vaccines and individuals. Therefore, some vaccines, such as for whooping cough, require boosters for adults whose immunity has worn off. Thirdly, some parents, although they are supportive of vaccination, do not arrange for all recommended vaccinations, due for example to forgetfulness, poverty or limited access to doctors. Fourthly, some parents prefer their children not to receive some or all vaccinations.

Campaigners for vaccination cannot do much about the first two categories — this is a task for scientists who search for improved vaccines — but can take action about the last two categories.

The Australian government, via federal and state health departments, promotes vaccination in a number of ways. One of the most important is authoritative endorsement of vaccination by the departments themselves and by expert advisers to the government, which include leading researchers [23]. Associated with this is authoritative endorsement by the Australian Medical Association [24]; medical practitioners have a high reputation compared to many other groups, such as politicians or corporations. Endorsement by respected expert authorities is central to what makes vaccination the dominant or standard position within the health field.

Most general practitioners accept this standard position; their recommendations to parents provide endorsement at another level, often more directly related to making decisions. Many people trust their personal doctors implicitly and would not think of challenging their advice, so endorsement at this level is highly influential.

In the face of the overwhelming endorsement of vaccination by health departments, researchers and doctors, there are a number of critics of vaccination, who argue that some or all vaccines are unnecessary or potentially harmful and that parents should have the choice to accept, delay or refuse some or all vaccines for their children [25]. In Australia, the most prominent vaccine-critical group was set up in the mid 1990s by Meryl Dorey. Its current name is the Australian Vaccination-risks Network (AVN). The group grew to have some 2000 members, hosted a large website and produced a glossy magazine.

In 2009, a pro-vaccination citizens’ group was set up; its current name is Stop the Australian (Anti)Vaccination Network (SAVN). From the beginning, SAVN’s explicit goal was to shut down the AVN. SAVN is a virtual group, primarily organised around its Facebook page, with thousands of friends, supplemented by blogs by individual SAVNers. SAVN apparently has no bank account, constitution, postal address, office bearers, formal meetings, minutes or other attributes of incorporated bodies.

SAVN introduced a new set of methods into the Australian vaccination struggle, mainly oriented around attempting to denigrate, harass and censor the AVN and other public critics of vaccination. SAVN initially made derogatory and unsupported claims about the AVN — for example that the AVN believed in a global conspiracy to implant mind control chips via vaccination — and targeted the AVN’s key figure Dorey for special contempt and abuse. SAVNers made dozens of complaints to government agencies about the AVN, serving as a form of harassment that distracted the AVN from its core activities. When Dorey organised talks or was reported in the media, SAVNers wrote letters of complaint in an attempt to have the talks cancelled and media coverage curtailed. Dorey and others in the AVN were sent pornography and received threats, though usually the senders remained anonymous [26, 27].

Meanwhile, social researchers supportive of vaccination have pursued a different path. A team led by Julie Leask of Sydney University has studied parents who have to make decisions about vaccination, classifying them into five categories: unquestioning acceptors, cautious acceptors, hesitants, late or selective vaccinators, and refusers. They then provided advice on dealing with parents in each category aimed at maximising acceptance of vaccination, based on providing information to parents in a respectful interaction. Rather than condemning reluctant parents or arguing with them, Leask et al. recommend listening to parents, engaging them in dialogue, and raising issues and asking questions according to the parents’ particular concerns ([28]; see also [29–31]). This approach has affinities with studies of risk communication by Dan Kahan and collaborators that suggest ways to promote vaccination by taking into account how individuals develop perceptions of risks [32, 33].

## Results

The three campaigning approaches for promoting vaccination in Australia can be used to illustrate ideal types. Each approach potentially incorporates several ideal types, as indicated in Table 2.

**Table 2:** Main ideal types expected in three approaches to vaccination campaigning

Principal group involved	Approach to vaccination campaigning	Main ideal types expected
Health departments; medical profession	Policy based on expert endorsement; inducements for parents	Publicity Valuing Endorsement Rewards Penalties
SAVN	Attacking vaccination critics	Censorship Devaluing Discrediting
Researchers	Engagement with parents	Valuing Arguments for

Table 2 illustrates that each of the three campaigning approaches is likely to involve more than one ideal type. Table 2 lists only the dominant ideal types expected for each approach, omitting types used less frequently. For example, SAVNers often cite evidence in favour of vaccination.

To illustrate a manifestation of the ideal types, it is convenient to examine submissions to a 2015 Australian Senate committee inquiry into proposed legislation to remove religious and conscientious objections to children’s vaccination for parents to receive certain welfare benefits [34]. Plans for this so-called “No jab, no pay” legislation triggered considerable public debate, and there were thousands of submissions to the inquiry. Of these, just three are scrutinised here: the Australian Medical Association [35], in the category of health authorities; SAVN [36]; and Leask and Wiley [37], in the category of researchers.

These three submissions are the best representatives of the three categories treated here. A comment is in order about related submissions. In the category of health authorities, Australian government health departments apparently did not make extensive submissions: the New South Wales Health Department made a one-page submission (#345) and the Royal Australasian College of Physicians made a short submission (#344). Other submissions in the same approach category as SAVN were those by the Australian Skeptics (#264), Friends of Science in Medicine (#316), and Northern Rivers Vaccination Supporters (#263). In the category of researchers, the submission by the Public Health Association of Australia (#317) is largely researcher-oriented.

When, in one of the three submissions chosen for analysis [35–37], a significant statement or an extended discussion reflecting a particular ideal campaigning type was found, a quote is used in Tables 3, 4 and 5 to illustrate the presence of the type.

**Table 3:** Quotes illustrating the presence of ideal campaigning types in the submission by the Australian Medical Association [35]

*Category*	Positive approach	Negative approach
*Information*		
*Attitude*		
*Arguments*	Arguments for: Herd immunity “provides additional protections in terms of decreasing the prevalence and circulation of disease …” (p. 1)	Arguments against: “The Bill does not mandate childhood immunisation.” (p. 3)
*Authorities*	Endorsement: “The Australian Medical Association (AMA) is a strong supporter of routine infant and child immunisation.” (p. 1)	
*Incentives*	Rewards: “Vaccination delivered according to the Immunisation Schedule is free for families.” (p. 3)	Penalties: “The AMA supports the removal of [conscientious exemption] as a measure to increase childhood immunisation rates.” (p. 2)

**Table 4:** Quotes illustrating the presence of ideal campaigning types in the submission by Stop the Australian (anti) Vaccination Network [36]

*Category*	Positive approach	Negative approach
*Information*		
*Attitude*		Devaluing: “… anti-vaccination advocacy on the part of people who have no regard for the truth or the health of their communities.” (p. 2)
*Arguments*	Arguments for: “All our citizens deserve protection from vaccine preventable disease.” (p. 4)	Arguments against: “Every piece of legislation presented so far to promote vaccination has at some time been accused of limiting freedom of speech …” (p. 5)
*Authorities*		Discrediting: “The AVN has not ceased operating and continues to attempt to mislead the public and legislators.” (p. 9)
*Incentives*		Penalties: “SAVN supports the removal of the ‘conscientious objection’ clause.” (p. 5)

**Table 5:** Quotes illustrating the presence of ideal campaigning types in the submission by researchers Leask and Wiley [37]

*Category*	Positive approach	Negative approach
*Information*		
*Attitude*	Valuing: “Quality engagement with a health professional is a much more ethical and satisfactory way to approach non-vaccinators than monetary sanctions.” (p. 4)	
*Arguments*	Arguments for: “All Australians have a responsibility to protect the vulnerable.” (p. 1)	
*Authorities*	Endorsement: “… vaccination is well supported by research …” (p. 1)	
*Incentives*		

Although Tables 3, 4 and 5 are not a complete representation of the themes in the three submissions, they do highlight distinct differences in approach. Most obviously, the SAVN submission [36] is dominated by negative approaches (Table 4) whereas the submission by researchers Leask and Wiley [37] is dominated by positive approaches (Table 5).

These tables are illustrative only, as several qualifications apply. First, submissions made to the inquiry may not fully represent methods used by health authorities, SAVN or researchers. Second, the submissions chosen for examination illustrating each approach are not necessarily typical of the overall approach. Third, submissions to an inquiry are a constrained type of health promotion; for example, they are less likely to contain publicity materials. Fourth, submissions may not articulate methods actually used in practice. For example, Dunlop and Stokes [36] do not mention SAVN’s efforts to censor the AVN.

Examination of Tables 3, 4 and 5 shows that the distribution of expected types itemised in Table 2 only partly overlaps with the distribution found in the submissions. This can be explained by the qualifications noted above; a more comprehensive survey of materials and actions would enable a better assessment of whether the expected types in Table 2 are appropriate.

Returning to Table 2, it would be possible to develop a more elaborate classification. Each principal group relies on others, who might be called agents, to implement its approach. For example, health departments and SAVN use the media to pursue their agendas; SAVN seeks to enrol government agencies as agents to attack critics; researchers aim to influence doctors and nurses to use their method of encouraging parents to vaccinate, while relying on government health departments to provide the context for their own efforts.

The tables can at most capture a snapshot of an ongoing process of campaigning. For example, the availability of the method of endorsement depends on the current state of play, in particular the willingness of experts to make a stand. In general terms, the campaigning methods depend on the current phase of a controversy, including the scientific evidence, key players and resources available.

Noting these limitations of the classification of approaches to campaigning illustrates a key point: it is difficult to analyse the effectiveness of an approach to campaigning, because there are so many complications and overlaps.

The promotion of vaccination in Australia overall has been highly effective, and promoters claim credit for the relatively low numbers of deaths and disabilities from vaccine-preventable diseases. However, this observation does nothing to distinguish the effectiveness of different approaches to campaigning.

## Discussion

Government health departments seem to imply that their current mix of methods to promote vaccination is optimal. For example, vaccination is free, which is an incentive, but vaccination is not mandatory except for some military personnel and health sector workers. However, health departments do not cite any rigorous studies to justify their choice of methods. For example, they do not give any empirical evidence that mandatory vaccination for health workers improves health outcomes.

SAVN commentators claim they have been effective, saying that their activities have curtailed the influence of the AVN by reducing its income and its credibility in media stories [38–40]. However, SAVN, despite a massive investment of effort in its campaign against the AVN, has never presented strong evidence that its campaign has increased vaccination rates or reduced the incidence or impact of vaccine-preventable illness.

Researchers into effectively promoting vaccination to parents can point to the results of various studies. However, whether widespread adoption of their approach by doctors and nurses would significantly improve vaccination rates and population health remains to be demonstrated.

There is ample evidence that vaccination can reduce morbidity and mortality for specific diseases [41, 42]. Citing such evidence is standard in vaccination campaigning, fitting within the category of ‘arguments for’. However, comparatively speaking, there is a dearth of evidence for the effectiveness of different approaches to promoting vaccination.

SAVN’s campaigning has been based on the assumption that the AVN’s activities had negatively affected vaccination rates and that discrediting and silencing the AVN would lead to improved health outcomes. A contrary view is that vaccine-critical groups have had little effect on vaccination rates, but rather are a response to concerns about vaccination that arise for other reasons, such as perceived adverse reactions of children to vaccinations [43]. This is compatible with the findings of a survey of AVN members showing that very few initially developed vaccine-critical views as a result of AVN materials; more commonly, members had concerns about vaccination and were attracted to the AVN because it provided a forum for these concerns [44]. From this perspective, trying to stifle vaccination critics is likely to have little impact on vaccination rates.

A possible proxy for the effectiveness of SAVN, in terms of its goal of promoting vaccination by discrediting and silencing critics, is the level of conscientious objection to vaccination. According to figures [45], the percentage of children whose parents sought conscientious objection increased every year from 2000 to 2014. The figure for 2015 is anomalous because the conscientious objection option was removed on 1 January 2016, reducing the incentive to apply in 2015. See Table 6. The increase from 2000 through 2014 may reflect parents’ increased awareness of the provision for conscientious objection [35, 37, 46]. The point here is that this trend predated the formation of SAVN and the furious struggle between SAVN and the AVN; the rate of increase, in percentage points per year, seems not to have changed substantially after SAVN became active beginning in 2009. Although this does not prove that SAVN has been ineffective, it is compatible with the view that vaccine-critical groups are more a product of parental concerns than a cause.

**Table 6:** Percentage of Australian children with conscientious objection to vaccination recorded at the end of calendar years [45]

Year	Percentage of children
1999	0.23
2000	0.41
2001	0.55
2002	0.67
2003	0.77
2004	0.86
2005	0.94
2006	1.03
2007	1.10
2008	1.20
2009	1.30
2010	1.36
2011	1.41
2012	1.46
2013	1.61
2014	1.77
2015	1.34

The aim here is not to assess the effectiveness of different approaches to promoting vaccination, but rather to illustrate the difficulties of assessing campaigning methods. It is useful here to return to the five types of complexities of campaigning noted in the introduction that make such assessments difficult and see how they are played out in the Australian vaccination struggle.

1. Controls. At least three different approaches to campaigning have been undertaken simultaneously in Australia, in most cases across the entire country, so there is no suitable control group. If infectious disease rates increase or decrease, it is hard to determine the cause, which in some cases may not be due to campaigning at all.
2. Outcomes. In the short term, SAVN has been effective in hindering AVN operations — for example, it ceased publishing its magazine — but it remains to be seen whether this is effective one or two decades hence. SAVN can point to changes in media coverage, with the AVN being given fewer favourable treatments, but whether this correlates with higher vaccination rates or lower disease rates is another question. Surrogate outcomes might be misleading if campaigning does not improve health.
3. Independent assessment. SAVN has been quick to claim success for its approaches, and health departments simply assume their policies are effective. However, there seem to be no independent studies of policies and approaches.
4. Causality. There are multiple causal factors influencing outcomes, including the effectiveness of vaccines, the general health of the community, attitudes of doctors, and information on the Internet, among others.
5. Agency. The targets of campaigning are active agents and may contest or resist efforts to change their behaviour. The AVN has come under sustained attack from SAVN and several government departments, yet has continued to exist and to develop innovative ways of circumventing censorship. Some parents may resent pressure to vaccinate, especially when doctors are arrogant or condemnatory. This is an argument against imposing financial and other penalties on parents whose children are not fully vaccinated: undue pressure may trigger greater resistance.

## Conclusion

The argument here is that campaigners on many health issues proceed with their preferred approach without much solid evidence that it is superior to alternative approaches. There is a certain irony in championing interventions to support evidence-based medicine while not having strong evidence to support the choice of how to promote the intervention. This seems inevitable due to the great difficulty in obtaining evidence at the level of approaches.

To help clarify campaigning options, ten ideal types were presented. In practice, approaches to campaigning, as the term “approaches” is used here, typically encompass several ideal types. Within an approach, it is more feasible to assess the effectiveness of campaigning methods. For example, if compliance is sought by rewards, then it is feasible to compare the effect of different rewards. However, when comparing different ideal-type methods or mixtures of methods, comparisons are more difficult.

Several reasons were presented as to why comparisons of campaigning approaches are more difficult than assessment of medical interventions. These include absence of controls, lack of independence of those doing assessments and the resistance of some subjects to interventions. A double-blind study of campaigning methods is hard to conceive.

An implication of this examination of campaigning methods is that claims about the effectiveness of particular strategies should be treated sceptically, especially when the claims are made by partisans involved in campaigning. Determining the effectiveness of campaigning approaches is vitally important, but it is wise to recognise the limitations of current knowledge.

## Abbreviations

AMA: Australian Medical Association
AVN: Australian Vaccination-risks Network
SAVN: Stop the Australian (Anti)Vaccination Network
